# Serum Vitamin D Levels and Its Relationship With Keloid, Acne or Hypertrophic Scar: A Two‐Sample Mendelian Randomization Study

**DOI:** 10.1111/jocd.70398

**Published:** 2025-08-21

**Authors:** Jiaqi Ji, Xinlong Shi, Enyu Tang, Jiazhen Zheng, Lei Liang

**Affiliations:** ^1^ Department of Ultrasound Aerospace Center Hospital Beijing China; ^2^ Department of Medicine 1 Friedrich‐Alexander University (FAU) Erlangen‐Nürnberg and Universitätsklinikum Erlangen Erlangen Bayern Germany; ^3^ Department of Gynecologic Oncology, National Cancer Center/National Clinical Research Center for Cancer/Cancer Hospital Chinese Academy of Medical Sciences and Peking Union Medical College Beijing China; ^4^ Bioscience and Biomedical Engineering Thrust, Systems Hub The Hong Kong University of Science and Technology (Guangzhou) Guangzhou Guangdong China

**Keywords:** Acne, Hypertrophic scar, Keloid, Mendelian randomization, Vitamin D

## Abstract

**Background:**

Previous studies reported that patients with keloid, acne, or hypertrophic scar (HS) had lower serum vitamin D levels compared to healthy controls. Whereas, these works failed to verify their causal relationships. Hence, we performed a Mendelian randomization (MR) aimed to investigate the causal relationship between vitamin D and keloid, acne, or HS.

**Methods:**

Utilizing summary datasets from genome‐wide association studies, we conducted a two‐sample MR analysis to explore the causal relationship between vitamin D and keloid, acne, or HS. The inverse‐variance weighted MR approach served as the primary analysis, with additional support from various sensitivity methods, including MR‐Egger, weighted median, simple mode, weighted mode, and MR‐PRESSO, to enhance the reliability of our findings.

**Results:**

From our MR analyses, no causal relationships were found between vitamin D level and keloid (OR: 1.15, 95% CI: 0.853–1.16, *p* = 0.36), acne (OR: 0.94, 95% CI: 0.66–1.30, *p* = 0.65) or HS (OR: 1.20, 95% CI 0.79–1.82, *p* = 0.40) formation. After eliminating outliers through MR‐PRESSO, no significant associations were found between serum vitamin D level and keloid, acne, as well as HS.

**Conclusion:**

Our findings, backed by rigorous IV selection and consistent outcomes across various MR approaches, suggest no causal link between serum 25(OH)D levels and keloid, acne, or HS. While vitamin D is involved in wound healing, its connection to these conditions may not be straightforward, emphasizing the need for further research.

## 1 Introduction

Vitamin D, a fat‐soluble prehormone, has important roles in lowering the likelihood of various chronic conditions and medical problems [1]. Vitamin D has different forms in our body, cholecalciferol (D_3_), ergocalciferol (D_2_), 25‐hydroxy vitamin D (25OHD), and calcitriol, and the amount of vitamin D is usually measured by 25OHD. The abundant amount of vitamin D is essential to maintain homeostasis of calcium and phosphorus, which regulate cellular proliferation and differentiation [2]. It also has a vital role in regulating inflammation by affecting the level of various inflammatory cytokines [3]. A randomized double‐blind controlled trial demonstrated that supplementation with vitamin D at doses of 1000 or 3000 IU/day would improve wound healing and decrease scar tissue thickness in hospitalized burn patients [4]. Another comparative study showed intralesional vitamin D is an alternative for keloid management for its efficiency and safety [5].

Keloid is a fibrotic skin disorder characterized by excessive collagen deposition. It is widely accepted that keloid formation and progression often stem from abnormal wound healing, triggering a fibroproliferative inflammatory response in which fibrin plays a crucial role [6]. The site of the lesion exhibits unique histologic characteristics: a large quantity of irregularly oriented, thickened transparent collagen fibers, and a large number of locally infiltrative inflammatory cells and cytokines [6]. Several studies reported that reduced vitamin D levels were found in patients with keloid, and intralesional vitamin D also has been used to treat keloid [7, 8]. However, a Korean cohort concluded preoperative serum vitamin D levels do not predict keloid recurrence [9]. Hence, is there a causal relationship between vitamin D levels and keloid formation?

Acne, an inflammatory skin condition associated with the pilosebaceous unit, affects over 20% of the global population, with the highest prevalence observed in individuals aged 16–24, reaching 28.3% [10]. Inflammation is a key factor in the onset, progression, and resolution of acne [11]. Wang et al. conducted a meta‐analysis and found that acne patients had lower serum 25(OH)D levels compared with the controls. But the exact role of vitamin D in acne still remains unclear [12].

A hypertrophic scar is an abnormal wound healing process marked by excessive collagen production in the skin, yet remaining within the original wound boundaries [13]. Many causes have been reported to have an effect on the pathophysiology of hypertrophic scar, such as genetic features, skin type, and surgery [14]. One study found that vitamin D deficiency may be the cause of the biomechanical properties of HS [15]. Patients with hypertrophic scar lack vitamin D, but vitamin D had no effect on the perceivability of the scar [14].

To investigate the causal link between vitamin D levels and pathological scars, we applied Mendelian Randomization (MR) analysis. MR utilizes naturally occurring genetic variations as instrumental variables (IVs), providing a study design comparable to randomized controlled trials, thereby strengthening causal inference. By selecting single nucleotide polymorphisms (SNPs) related to vitamin D as IVs from published genome‐wide association studies (GWAS), we take advantage of the random distribution of these SNPs to strengthen cause‐and‐effect interpretations. Therefore, this MR study establishes a foundation for further subsequent intervention‐based and treatment‐oriented studies.

## 2 Methods

### 2.1 Study Design

**FIGURE 1:**
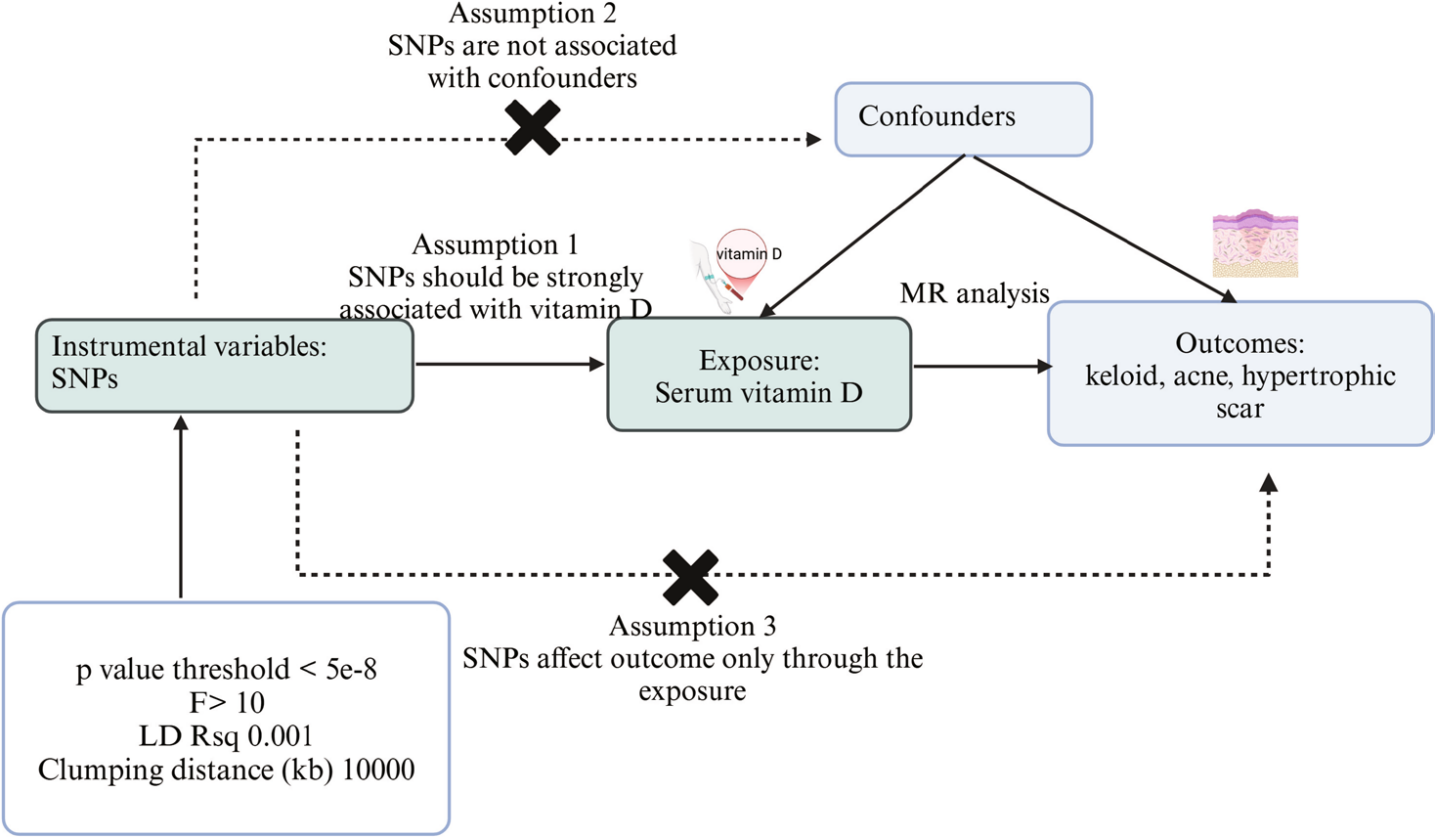
Workflow of MR study revealing causal relationship between serum 25(OD)D level and keloid, acne, and hypertrophic scar. MR, Mendelian randomization.

According to the Strengthening the Reporting of Observational Studies in Epidemiology using Mendelian Randomization (STROBE‐MR) statement [16], we designed a visual framework of our study (Figure 1). To reduce the potential bias caused by population stratification, both the exposure and outcome groups were primarily restricted to individuals of European ancestry. In this study, the GWAS Catalog (https://www.ebi.ac.uk/gwas/) and the IEU OpenGWAS project (https://gwas.mrcieu.ac.uk/) were searched with vitamin D to find the most recent large‐scale sample size and SNPs. Like Qiu et al. [17], this study only concentrated on the European population and analyzed data based on serum levels of vitamin D without supplement intake to ensure more accurate levels of vitamin D in the body and reduce bias. No Artificial Intelligence Generated Content was used in this study.

### 2.2 Exposure and Outcomes Database

Firstly, 417 580 European participants had both the distribution of 25OHD concentration and genome‐wide genotypes selected as exposures in our study [18]. Then we selected the latest large‐scale GWAS of keloid, acne, or hypertrophic scar. Detailed information is demonstrated in Table 1.

**TABLE 1:** The detailed information on the GWAS used in this study.

	Exposure	Outcomes		
	Serum 25‐Hydroxyvitamin D levels	Keloid	Acne	Hypertrophic scar
Dataset	ebi‐a‐GCST90000618	ebi‐a‐GCST90018874	finn‐b‐L12_ACNE	finn‐b‐L12_HYPETROPHICSCAR
PMID	32 242 144	34 594 039	/	/N
Year	2020	2021	2021	2021
Category	NA	NA	Binary	Binary
Subcategory	NA	NA	NA	NA
Population	European	European	European	European
Sex	NA	NA	Males and females	Males and females
ncase	NA	668	1299	766
ncontrol	NA	481 244	211 139	207 482
Sample size	496 946	481 912	/	/
Number of SNPs	6 896 093	24 197 210	16 380 454	16 380 443
Author	Revez JA	Sakaue S	NA	NA
Consortium	NA	NA	NA	NA
Build	HG19/GRCh37	HG19/GRCh37	HG19/GRCh37	HG19/GRCh37

For the MR analysis, SNPs selected as instrumental variables (IVs) should meet the following three critical assumptions. The first one is the association assumption; it means all selected IVs must be strongly correlated with vitamin D. The second one is the exclusivity assumption, in which outcomes should not be affected by IVs. The independent assumption is the last criterion, and IVs must be independent of any potential confounders [19].

### 2.3 IVs Selection

The process of selecting IVs is as follows: [1] identifying SNPs significantly associated with vitamin D levels (*p* < 5 × 10^−8^); [2] ensuring independence by removing those in linkage disequilibrium (LD) (*r* ^2^ = 0.001, distance = 10 000 KB); and [3] integrating relevant GWAS summary statistics for the outcome. We calculated *F*‐statistics to exclude weak IVs. The variance (*R* ^2^) for each SNP was computed as *R* ^2^ = 2 × EAF × (1−EAF) × *β* ^2^, and the *F*‐statistic as *F* = *R* ^2^ (*N*−2)/(1−*R* ^2^), where EAF is the effect allele frequency, *β* is the effect estimate in the exposure GWAS, and *N* refers to corresponding sample size. SNPs would be removed if the *F*‐statistic < 10 were found [20].

### 2.4 MR Analysis of Serum Vitamin D Level on Keloid, Acne and Hypertrophic Scar

The causal association estimates were calculated using the IVW method, with results expressed as effect size (*β*) and a 95% confidence interval (CI) for vitamin D levels, and as odds ratios (OR) with a 95% CI for keloid, acne, or HS. Based on heterogeneity assessment outcomes, causal estimations were derived using either inverse‐variance weighted (IVW) random‐effects models (when significant heterogeneity was detected) or IVW fixed‐effects approaches (under homogeneous conditions). A *p* value below 0.05 was considered indicative of statistical significance.

### 2.5 Sensitivity Analysis

All Mendelian randomization analyses were conducted in R studio (version 4.4.3). SNP heterogeneity was quantitatively evaluated through Cochran's *Q* test, with the statistical significance threshold set at *α* = 0.05. Horizontal pleiotropy was systematically examined through MR‐Egger regression intercept analysis, where a nonsignificant deviation from null (*p* ≥ 0.05) suggested the absence of directional pleiotropic effects. To ensure analytical robustness, we implemented MR‐PRESSO methodology for simultaneous outlier detection and pleiotropy evaluation, complemented by leave‐one‐out sensitivity analyses to verify result consistency through the sequential exclusion of individual genetic instruments. The analytical workflow employed TwoSampleMR (version 0.6.11) and MR‐PRESSO (version 1.0) packages, maintaining methodological consistency across all investigations.

## 3 Results

### 3.1 Selection of IVs

The causal relationship between vitamin D and keloid, acne, and hypertrophic scar was conducted using two‐sample MR analysis. After selection by R studio, 117 SNPs strongly associated with vitamin D were identified (Table S1). All genetic instruments demonstrated strong instrument strength (*F*‐statistics > 10), with the minimum *F*‐statistic reaching 30.46, confirming the absence of weak instrument bias and reinforcing the reliability of the selected variants as valid instrumental variables. Less than 3 SNPs were removed during MR analysis (Tables S2–S4).

### 3.2 Vitamin D and Keloid

**FIGURE 2:**
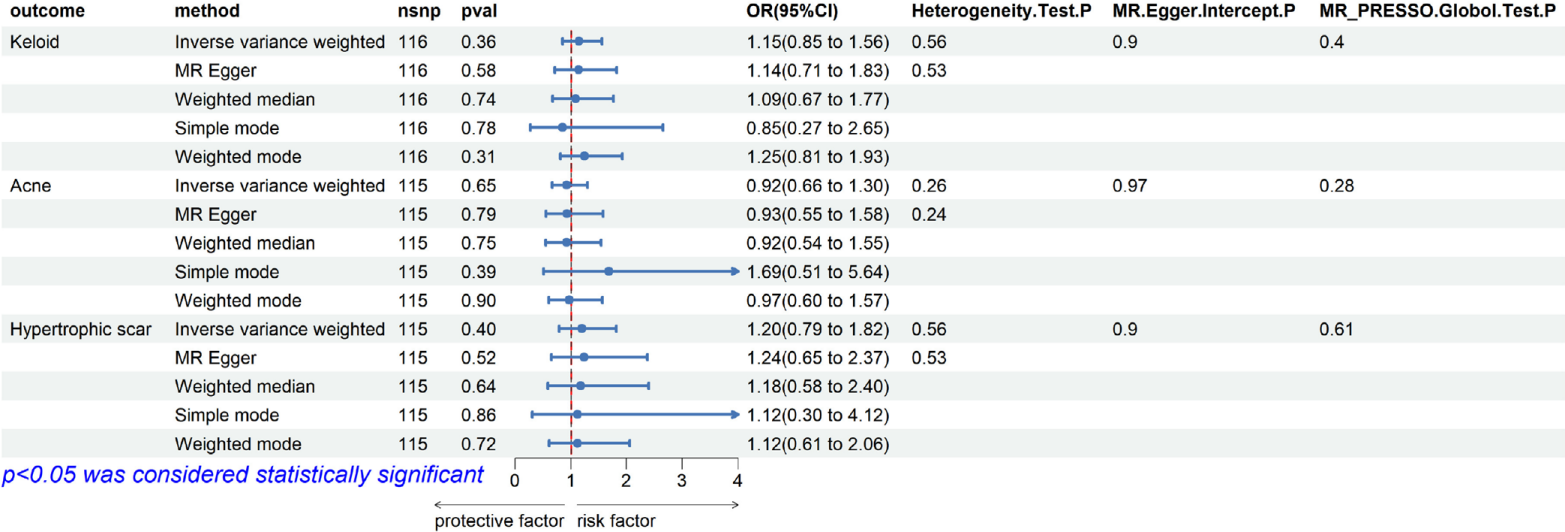
MR analysis on the association between serum 25(OD)D level and keloid, acne, and hypertrophic scar.

**FIGURE 3:**
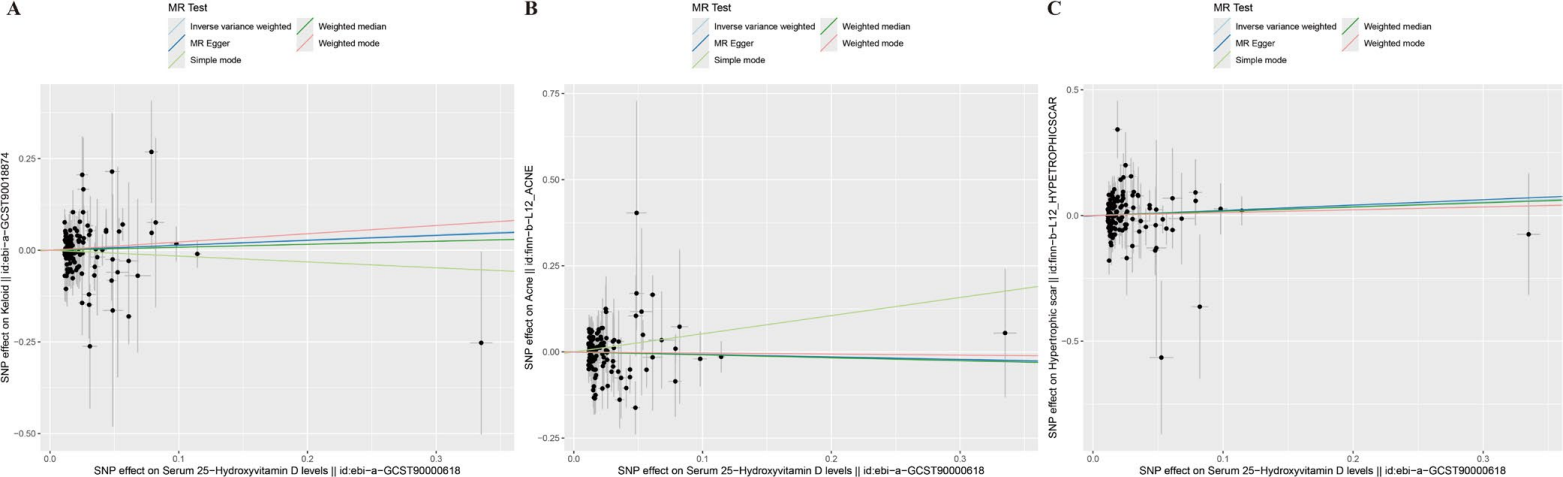
Scatter plot correlation between vitamin D and keloid, acne, and hypertrophic scar risk across different SNPs. (A) keloid; (B) acne; and (C) hypertrophic scar.

No significant relationship between vitamin D and keloid was found by IVW method (OR: 1.15, 95% CI: 0.853–1.16, *p* = 0.36). Similarly, the weighted median (*p* = 0.74) and weighted mode (*p* = 0.31) approaches provided no evidence of a causal relationship between vitamin D and keloid (Figure 2). Scatter plot showing the positive correlation between vitamin D and keloid risk across different SNPs (Figure 3A). Furthermore, there is no obvious heterogeneity (*p* for Cochran's *Q* test = 0.39) or horizontal pleiotropy (*p* for Egger intercept = 0.97, Table S6). MR‐PRESSO analysis also did not identify any meaningful outlier SNPs or indications of pleiotropy (*p* for the global test = 0.40). The leave‐one‐out sensitivity analysis confirmed the stability of the results, as excluding any single SNP did not alter the main findings (Appendix S1). Collectively, these multivariable MR investigations provide consistent evidence against vitamin D's protective role in keloid formation, with methodological coherence maintained across sensitivity frameworks.

### 3.3 Vitamin D and Acne

The primary IVW analysis demonstrated nonsignificant risk reduction for vitamin D in acne pathogenesis (OR = 0.94, 95% CI = 0.66–1.30, *p* = 0.65). This null association was corroborated by secondary MR approaches, including weighted median (*p* = 0.27) and weighted mode (*p* = 0.25) estimators (Figure 2). Although SNP‐level effect estimates exhibited nominally positive associations in multivariant analysis (Figure 3B), comprehensive methodological validation revealed: (1) Instrumental homogeneity (Cochran's *Q p* = 0.24); (2) No directional pleiotropy (MR‐Egger intercept *p* = 0.97; Table S6); (3) MR‐PRESSO detected neither influential outliers (global test *p* = 0.28) nor systemic pleiotropic bias. Leave‐one‐out analysis, which excluded variants sequentially, ensured the stability of the effect estimate across all permutations (Appendix S1). Overall, these results suggest that vitamin D does not reduce the risk of acne formation, and the consistency of the result was reinforced through multiple sensitivity analyses.

### 3.4 Vitamin D and Hypertrophic Scar

As Figure 2 demonstrated, the OR of 25(OD)D in hypertrophic scar was 1.20, with a 95% CI of 0.79–1.82 (*p* = 0.40). Methodological consistency was observed across supplementary MR approaches, with weighted median (*p* = 0.64) and weighted mode (*p* = 0.72) estimators similarly failing to establish causal inference between circulating vitamin D concentrations and hypertrophic scar pathogenesis (Figure 2). A positive but not significant correlation was detected between vitamin D and hypertrophic scar risk across different SNPs (Figure 3C). No significant heterogeneity (*p* = 0.53) and horizontal pleiotropy (*p* = 0.90, Table S6) were found in sensitivity analysis. Additionally, no significant outlier SNPs or indications of pleiotropy were identified (*p* for global test = 0.61). Exclusion of any single SNP did not materially alter the estimates, which confirmed the stability of results (Appendix S1). Taken together, these findings provide no genetic evidence supporting a protective effect of vitamin D against keloid formation.

## 4 Discussion

Keloid, acne, and hypertrophic scar are inflammatory dermatological conditions, and these pathological scars are not only an aesthetic issue but also affect the patients' quality of life [6, 21, 22, 23]. A previous Mendelian randomization study revealed that high vitamin D status may fight against chronic inflammation [24]. However, no study about the causal relationship between vitamin D and keloid, acne, or hypertrophic scar was conducted. Therefore, this MR analysis attempts to explore the potential causal relationship between serum 25(OH)D level and keloid, acne, and hypertrophic scar. Applying genetic variation, we aimed to clarify the relationship within a two‐sample MR research, where serum 25(OH)D levels were used as exposure and keloid, acne, and hypertrophic scar were used as outcomes. No evidence of genetic associations was found between these variables, suggesting serum 25(OH)D levels could not decrease the risk of formation of keloid, acne, or hypertrophic scar.

A U‐shaped relation was found between 25(OH)D and C‐reactive protein, and also an inverse association between 25(OH)D and fibrinogen was found [25], which confirmed a potential role of 25(OH)D and chronic inflammation. Clinical studies also found that keloid patients had significantly lower 25(OH)D versus healthy controls [26, 27]. These results implied the potential relationship of vitamin D and keloid. Based on these findings, intralesional vitamin D has been used to treat keloid in clinical trials [5, 7] and it has been proven to be a reliable and efficient approach in the management of keloid. Whereas a Korean retrospective cohort found preoperative vitamin D levels do not predict keloid recurrence. Vitamin D's effect might be overshadowed by more dominant factors influencing keloid recurrence [9]. The immunomodulatory properties of vitamin D are mediated through VDR‐expressing immune cell populations, orchestrating cytokine homeostasis by suppressing proinflammatory cytokine biosynthesis while potentiating anti‐inflammatory mediator synthesis through receptor‐dependent signaling mechanisms [8]. This means not only the serum vitamin 25(OH)D level but the VDR level could influence the anti‐inflammatory effect of vitamin D. Keloid fibroblasts (KFs) have been confirmed to express VDRs, and the expression of TGF‐β1‐induced collagen type I, fibronectin, and α‐smooth muscle actin were suppressed in KFs cultured with 1,25 D in an in vitro analysis [28]. In mice lacking the vitamin D receptor (VDR‐null), the expression of resin and plasma angiotensin II were markedly elevated, resulting in the development of hypertension and cardiac hypertrophy [29]. Therefore, it is hypothesized that the reduced levels of vitamin D and VDR observed in keloids may weaken the regulatory effect of vitamin D on the renin–angiotensin system (RAS), thereby promoting the development of hypertension and potentially increasing the risk of keloid formation [30]. However, due to the limited number of strong SNPs related to vitamin D‐binding protein (*n* = 2), we failed to detect the causal relationship of vitamin D‐binding protein and keloid. Studies with large enough sample size should be designed to research this correlation.

Fibroblast‐driven collagen overproduction underlies hypertrophic scarring through dysregulated wound repair mechanisms. Due to the anti‐inflammation effect of vitamin D, studies were conducted to compare the vitamin D plasma levels of HS patients and controls [15, 31]. In a Turkey cohort, authors examined whether scar outcomes could be improved by vitamin D supplementation [14]. However, vitamin D monotherapy demonstrated no therapeutic efficacy on the severity of HS. In a study of keloid or hypertrophic scar‐affected patients in the UK biobank, vitamin D deficiency was a risk factor for keloid or hypertrophic scar in Asian participants (OR = 2.24, 95% CI: 1.26–3.97, *p* = 0.006) rather than black participants (OR = 0.88, 95% CI: 0.37–2.08, *p* = 0.77) or white participants (OR = 1.32, 95% CI: 0.85–2.07, *p* = 0.22) [32]. Keloid and HS are more common in dark‐skinned people, Asians, and Africans than in other ethnicities [13, 33]. As a result, ethnicity should be taken into consideration when studying the relationship between vitamin D and keloid or HS. In our MR analysis, our participants were Europeans, and no causal relationship was found. Hence, the relationship between vitamin D and HS in other populations should be conducted in further studies.

As a chronic inflammatory disease of skin, the link between acne and vitamin D was also researched to evaluate vitamin D supplementation therapeutic potential [22, 34, 35]. A meta‐analysis found patients with acne had significantly lower serum vitamin D, and vitamin D supplement could reduce the inflammation, but most of the studies included were conducted in Asia [36]. Our MR result shows no significant difference was found between 25(OH)D level and acne (OR: 0.94, 95% CI: 0.66–1.30, *p* = 0.65) without any significant heterogeneity or pleiotropy. Adolescents are the primary population affected by acne; hence, the relationship between vitamin D and acne in adolescents should be studied in the future.

Dark‐skinned populations, especially those of African or Asian ancestry, are more prone to developing keloids and hypertrophic scars [37, 38]. The development and progression of keloids are closely related to genetic factors. Compared with the White population, Black or African American individuals demonstrated decreased nuclear localization of VDR, which is thought to increase the risk of keloid formation [39]. Baseline concentrations of provitamin D appear consistent across ethnicities; however, UV‐induced vitamin D_3_ synthesis differs by skin pigmentation, with lighter‐skinned individuals exhibiting more efficient production than those with darker skin tones [40]. Hence, given the known differences in Vitamin D metabolism and genetic factors across ethnic groups, future investigations in Asian and African populations are necessary to validate the causal relationship between Vitamin D and keloid or HS.

This study has some strengths. The relationship of serum vitamin D levels and the risk of keloid, acne, or hypertrophic scar conducted by comprehensive MR analysis is the obvious advantage of this study. Secondly, the exposure and outcome summary data were selected from independent GWAS sources to avoid confounding bias arising from potentially overlapped samples. Finally, the conclusions were drawn through large‐scale sample sizes and robust methodology. Four methodological limitations warrant acknowledgement: (1) European‐centric GWAS data constrain cross‐population generalizability; (2) Analytical framework restricted to linear dose–response dynamics, neglecting potential nonlinear threshold effects; (3) Omission of vitamin D‐binding protein assessments in dermatosis pathophysiology; (4) Although no significant pleiotropy was detected in this study, the integration of multi‐omics data (e.g., transcriptomic profiles) in future investigations may help strengthen the validation of instrument specificity. Future investigations require mechanistic studies with ethnic diversity sampling and vitamin D metabolic pathway triangulation through preclinical models, clinical cohorts, and population‐specific analyses.

## 5 Conclusion

This Mendelian randomization study performed comprehensive analyses of genetically predicted serum vitamin D concentrations across three fibroproliferative dermatoses (keloids, acne, and hypertrophic scarring). Analytical results consistently demonstrated no genetic associations across all outcomes (*p* > 0.05) in European populations based on MR analyses. Despite several limitations, this study offers valuable insights into the association between vitamin D and keloid, acne, or hypertrophic scar in European populations, calling for the need for further research in diverse populations and laying the groundwork for future interventional studies targeting these conditions.

## Author Contributions

Jiaqi Ji and Xinlong Shi carried out the studies, participated in collecting data, analysis and drafted the manuscript. Enyu Tang and Jiazhen Zheng provided support to conduct MR analyze. Xinlong Shi and Lei Liang revised the original manuscript. All authors read and approved the final manuscript.

## Ethics Statement

Since this study used publicly available data, no ethical approval was required.

## Consent

All data used in this Mendelian Randomization study were obtained from publicly available genome‐wide association studies (GWAS) with existing ethics approval and informed consent from participants. No individual‐level data were used, and therefore no additional ethical approval was required for this analysis.

## Conflicts of Interest

The authors declare no conflicts of interest.

## Supporting information


**Table S1:** jocd70398‐sup‐0001‐TablesS1‐S6.xlsx.

**Appendix S1:** jocd70398‐sup‐0001‐AppendixS1.pdf.

## Data Availability

The data that support the findings of this study are available on request from the corresponding author. The data are not publicly available due to privacy or ethical restrictions.
