# Supplementary material for: Serum Vitamin D Levels and Its Relationship With Keloid, Acne or Hypertrophic Scar: A Two‐Sample Mendelian Randomization Study
Source: J Cosmet Dermatol. 2025 Aug 21;24(8):e70398. doi: 10.1111/jocd.70398 (PMC12368766; doi:10.1111/jocd.70398)
Supplement: Supplementary file 2 — Appendix S1: jocd70398‐sup‐0001‐AppendixS1.pdf. [file JOCD-24-e70398-s001.pdf]

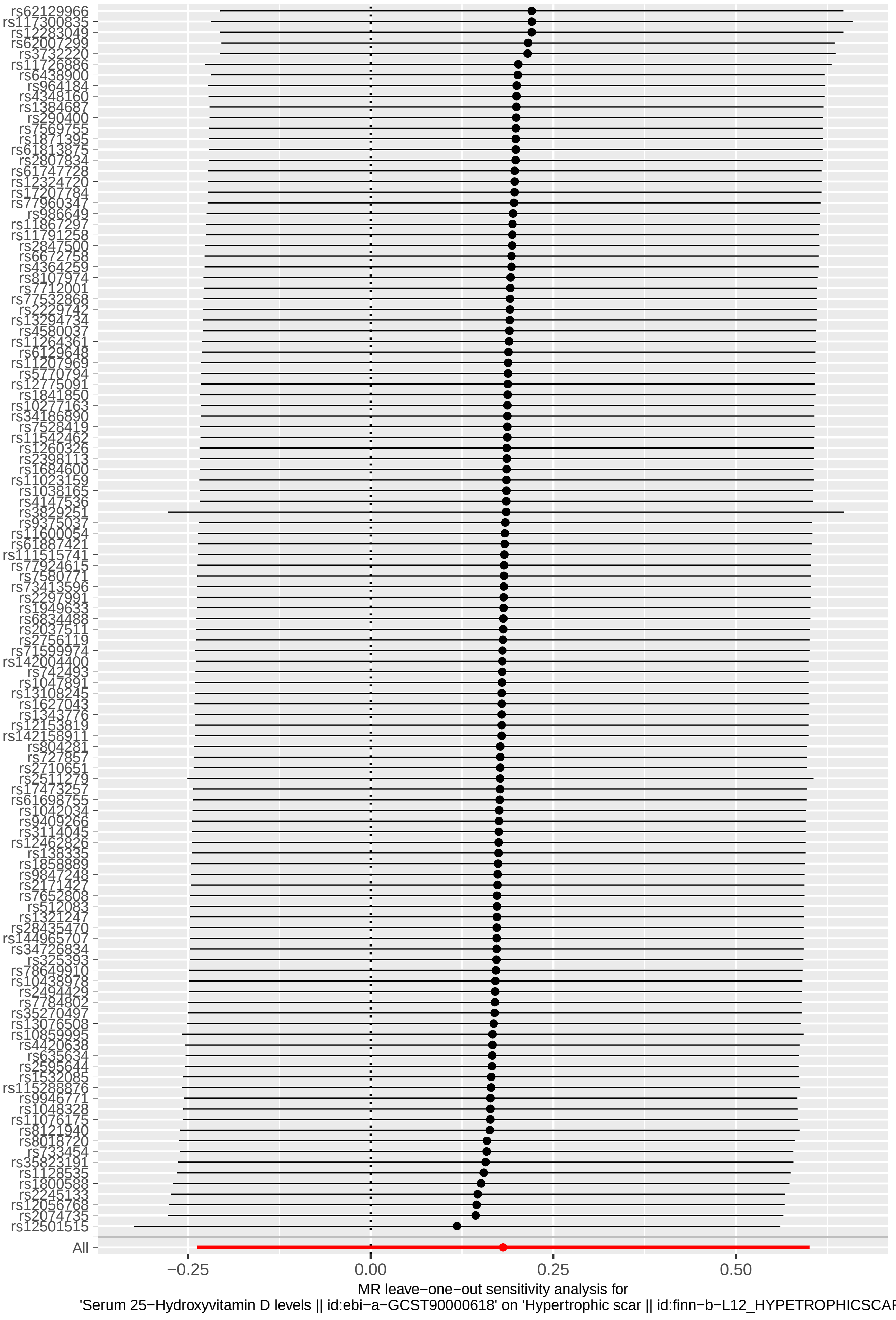
rs62129966
rs117300835
rs12283049
rs62007299
rs3732220
rs11726886
rs6438900
rs964184
rs4348160
rs1384687
rs290400
rs7569755
rs1871395
rs61813875
rs2807834
rs61747728
rs12324720
rs17207784
rs77960347
rs986649
rs11867297
rs11791258
rs2847500
rs6672758
rs4364259
rs8107974
rs7712001
rs77532868
rs2229742
rs13294734
rs4580037
rs11264361
rs6129648
rs11207969
rs5770794
rs12775091
rs1841850
rs10277163
rs34186890
rs7528419
rs11542462
rs1260326
rs2398113
rs1684600
rs11023159
rs1038165
rs4147536
rs3829251
rs9375037
rs11600054
rs61887421
rs111515741
rs77924615
rs7580771
rs73413596
rs2297991
rs1949633
rs6834488
rs2037511
rs2756119
rs71599974
rs142004400
rs742493
rs1047891
rs13108245
rs1627043
rs1343776
rs12153819
rs142158911
rs804281
rs727857
rs2710651
rs2511279
rs17473257
rs61698755
rs1042034
rs9409266
rs3114045
rs12462826
rs138335
rs1858889
rs9847248
rs2171427
rs7652808
rs512083
rs1321247
rs28435470
rs144965707
rs34726834
rs325393
rs78649910
rs10438978
rs2494429
rs7784802
rs35270497
rs13076508
rs10859995
rs4420638
rs635634
rs2595644
rs1532085
rs115288876
rs9946771
rs1048328
rs11076175
rs8121940
rs8018720
rs733454
rs35823191
rs1128535
rs1800588
rs2245133
rs12056768
rs2074735
rs12501515
All
−0.25
0.00
0.25
0.50
MR leave−one−out sensitivity analysis for
'Serum 25−Hydroxyvitamin D levels || id:ebi−a−GCST90000618' on 'Hypertrophic scar || id:finn−b−L12_HYPETROPHICSCAR

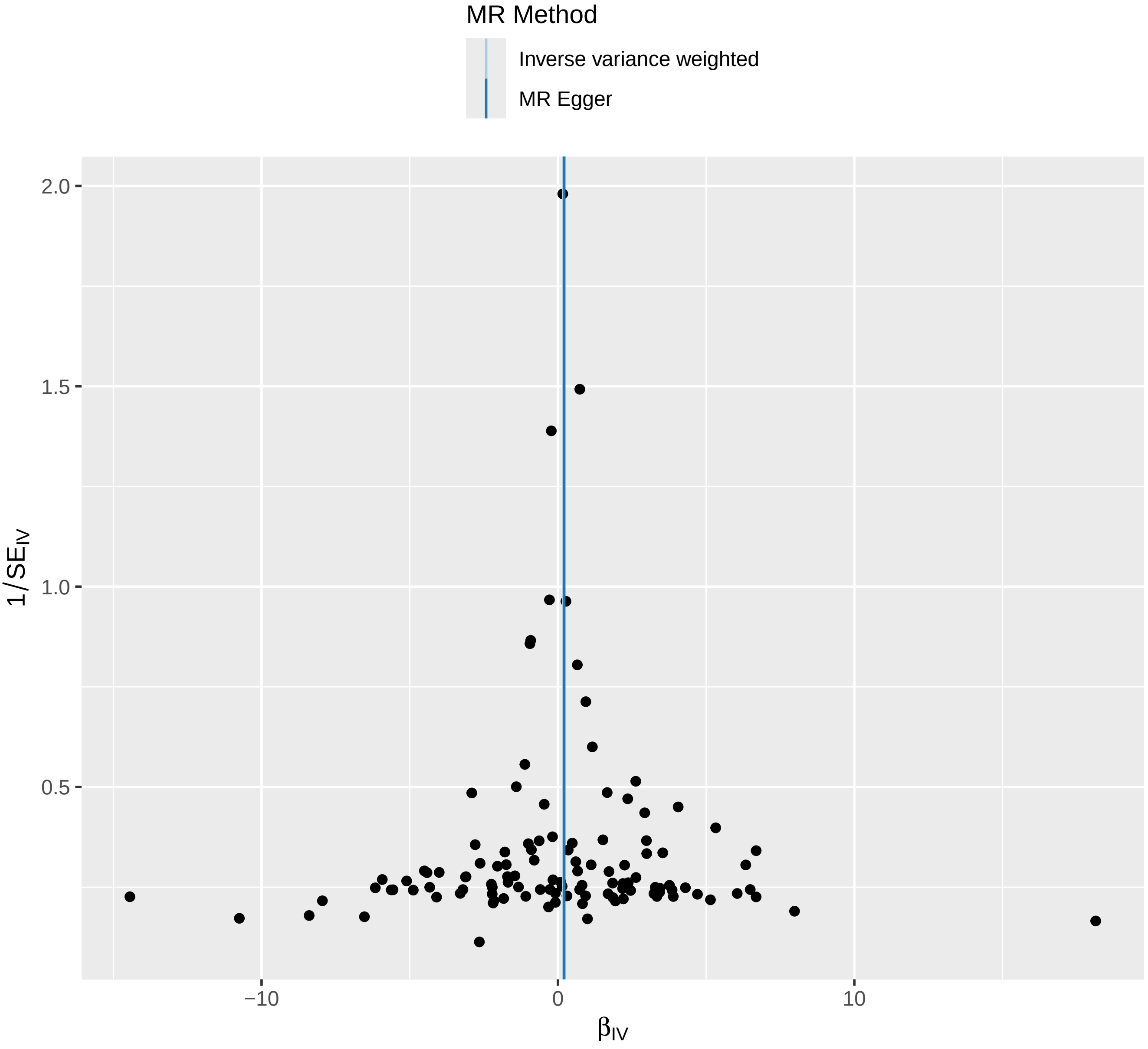

MR Method
Inverse variance weighted
MR Egger
2.0
1.5
1.0
0.5
$1/SE_{IV}$
−10
0
10
β_{IV}

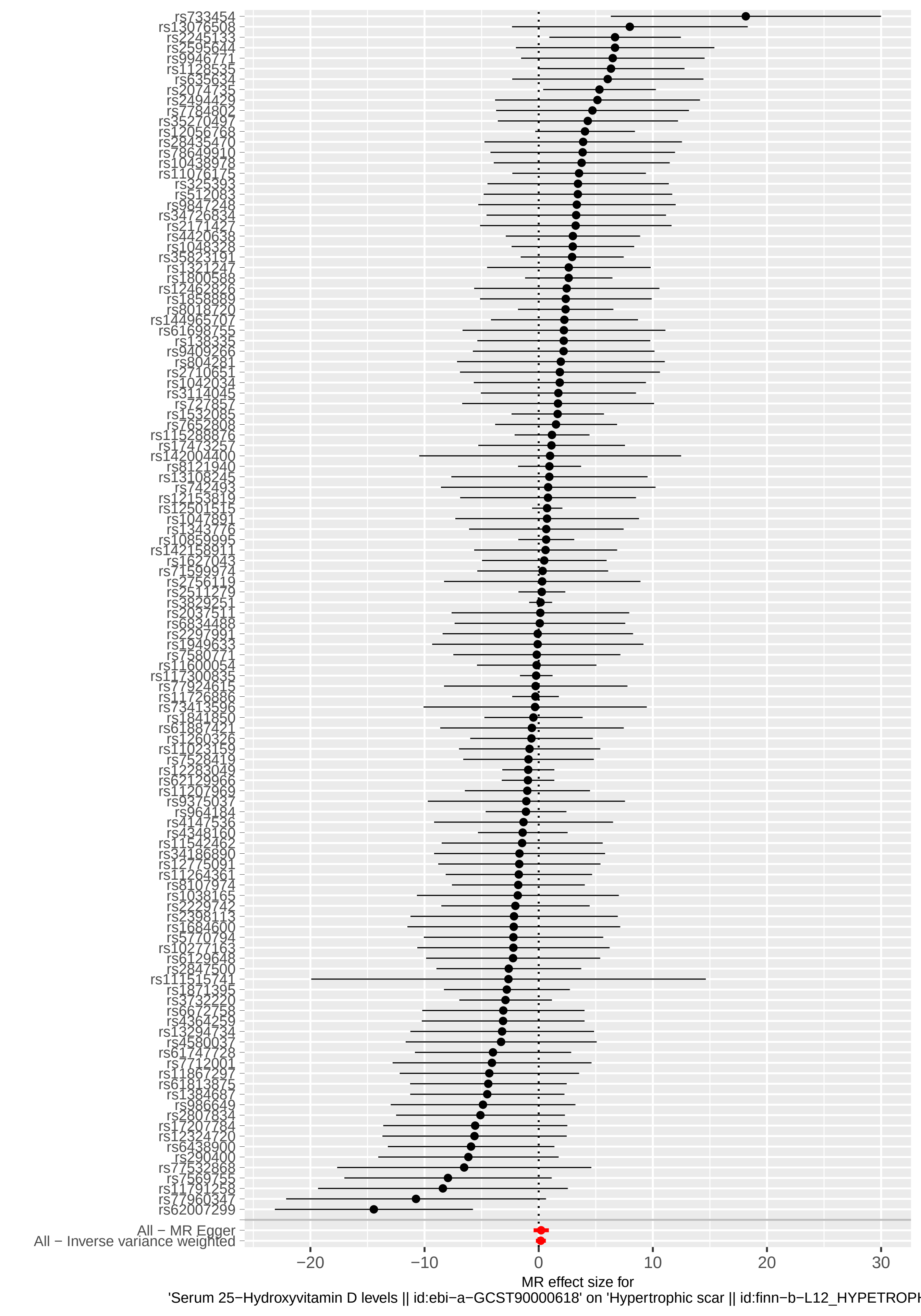
rs733454
rs13076508
rs2245133
rs2595644
rs9946771
rs1128535
rs635634
rs2074735
rs2494429
rs7784802
rs35270497
rs12056768
rs28435470
rs78649910
rs10438978
rs11076175
rs325393
rs512083
rs9847248
rs34726834
rs2171427
rs4420638
rs1048328
rs35823191
rs1321247
rs1800588
rs12462826
rs1858889
rs8018720
rs144965707
rs61698755
rs138335
rs9409266
rs804281
rs2710651
rs1042034
rs3114045
rs727857
rs1532085
rs7652808
rs115288876
rs17473257
rs142004400
rs8121940
rs13108245
rs742493
rs12153819
rs12501515
rs1047891
rs1343776
rs10859995
rs142158911
rs1627043
rs71599974
rs2756119
rs2511279
rs3829251
rs2037511
rs6834488
rs2297991
rs1949633
rs7580771
rs11600054
rs117300835
rs77924615
rs11726886
rs73413596
rs1841850
rs61887421
rs1260326
rs11023159
rs7528419
rs12283049
rs62129966
rs11207969
rs9375037
rs964184
rs4147536
rs4348160
rs11542462
rs34186890
rs12775091
rs11264361
rs8107974
rs1038165
rs2229742
rs2398113
rs1684600
rs5770794
rs10277163
rs6129648
rs2847500
rs111515741
rs1871395
rs3732220
rs6672758
rs4364259
rs13294734
rs4580037
rs61747728
rs7712001
rs11867297
rs61813875
rs1384687
rs986649
rs2807834
rs17207784
rs12324720
rs6438900
rs290400
rs77532868
rs7569755
rs11791258
rs77960347
rs62007299
All − MR Egger
All − Inverse variance weighted
−20
−10
0
10
20
30
MR effect size for
'Serum 25−Hydroxyvitamin D levels || id:ebi−a−GCST90000618' on 'Hypertrophic scar || id:finn−b−L12_HYPETROPH

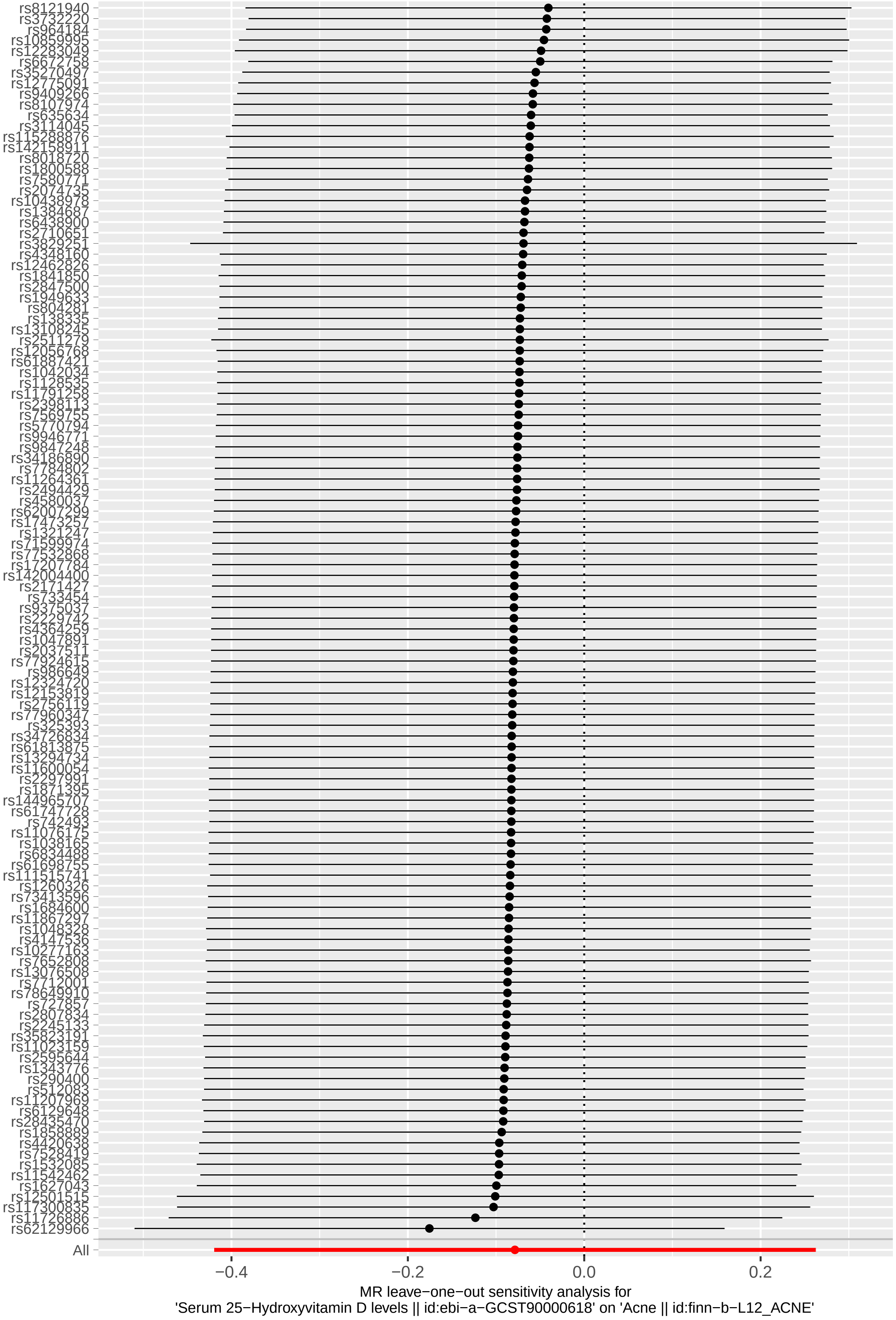
rs8121940
rs3732220
rs964184
rs10859995
rs12283049
rs6672758
rs35270497
rs12775091
rs9409266
rs8107974
rs635634
rs3114045
rs115288876
rs142158911
rs8018720
rs1800588
rs7580771
rs2074735
rs10438978
rs1384687
rs6438900
rs2710651
rs3829251
rs4348160
rs12462826
rs1841850
rs2847500
rs1949633
rs804281
rs138335
rs13108245
rs2511279
rs12056768
rs61887421
rs1042034
rs1128535
rs11791258
rs2398113
rs7569755
rs5770794
rs9946771
rs9847248
rs34186890
rs7784802
rs11264361
rs2494429
rs4580037
rs62007299
rs17473257
rs1321247
rs71599974
rs77532868
rs17207784
rs142004400
rs2171427
rs733454
rs9375037
rs2229742
rs4364259
rs1047891
rs2037511
rs77924615
rs986649
rs12324720
rs12153819
rs2756119
rs77960347
rs325393
rs34726834
rs61813875
rs13294734
rs11600054
rs2297991
rs1871395
rs144965707
rs61747728
rs742493
rs11076175
rs1038165
rs6834488
rs61698755
rs111515741
rs1260326
rs73413596
rs1684600
rs11867297
rs1048328
rs4147536
rs10277163
rs7652808
rs13076508
rs7712001
rs78649910
rs727857
rs2807834
rs2245133
rs35823191
rs11023159
rs2595644
rs1343776
rs290400
rs512083
rs11207969
rs6129648
rs28435470
rs1858889
rs4420638
rs7528419
rs1532085
rs11542462
rs1627043
rs12501515
rs117300835
rs11726886
rs62129966
All
−0.4
−0.2
0.0
0.2
MR leave−one−out sensitivity analysis for
'Serum 25−Hydroxyvitamin D levels || id:ebi−a−GCST90000618' on 'Acne || id:finn−b−L12_ACNE'

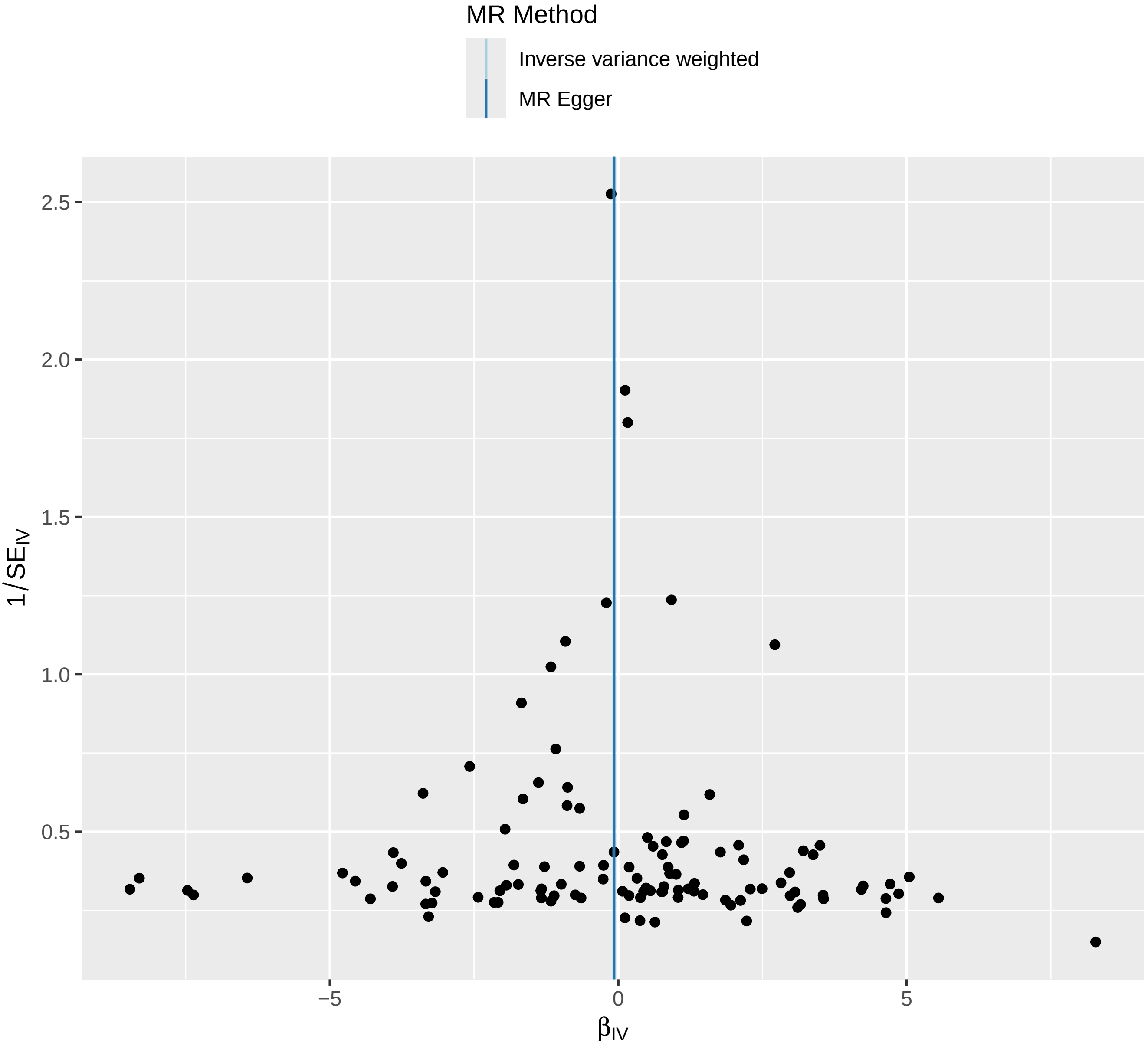
MR Method
Inverse variance weighted
MR Egger
2.5
2.0
1.5
1.0
0.5
$1/SE_{IV}$
−5
0
5
β_{IV}

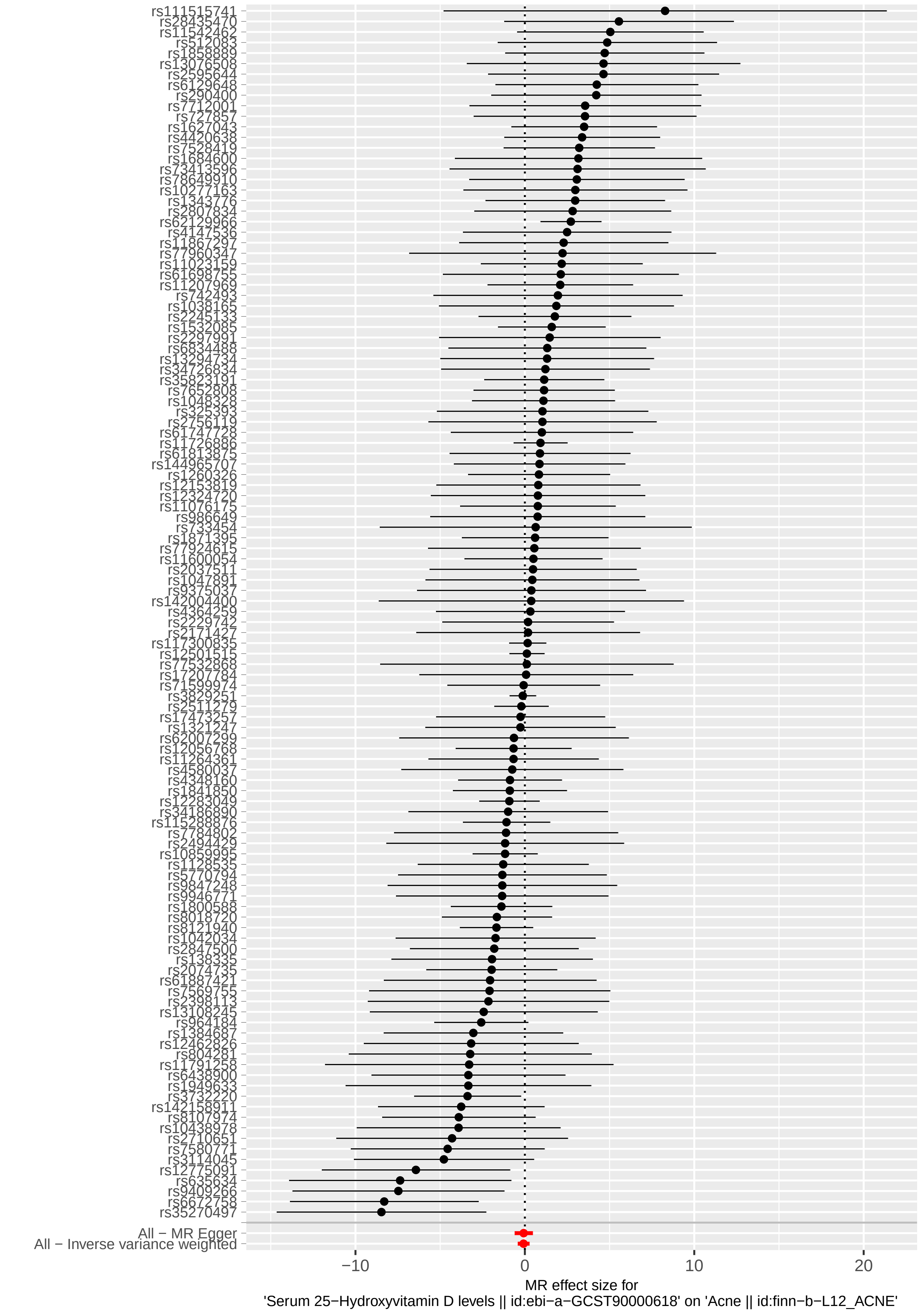
rs111515741
rs28435470
rs11542462
rs512083
rs1858889
rs13076508
rs2595644
rs6129648
rs290400
rs7712001
rs727857
rs1627043
rs4420638
rs7528419
rs1684600
rs73413596
rs78649910
rs10277163
rs1343776
rs2807834
rs62129966
rs4147536
rs11867297
rs77960347
rs11023159
rs61698755
rs11207969
rs742493
rs1038165
rs2245133
rs1532085
rs2297991
rs6834488
rs13294734
rs34726834
rs35823191
rs7652808
rs1048328
rs325393
rs2756119
rs61747728
rs11726886
rs61813875
rs144965707
rs1260326
rs12153819
rs12324720
rs11076175
rs986649
rs733454
rs1871395
rs77924615
rs11600054
rs2037511
rs1047891
rs9375037
rs142004400
rs4364259
rs2229742
rs2171427
rs117300835
rs12501515
rs77532868
rs17207784
rs71599974
rs3829251
rs2511279
rs17473257
rs1321247
rs62007299
rs12056768
rs11264361
rs4580037
rs4348160
rs1841850
rs12283049
rs34186890
rs115288876
rs7784802
rs2494429
rs10859995
rs1128535
rs5770794
rs9847248
rs9946771
rs1800588
rs8018720
rs8121940
rs1042034
rs2847500
rs138335
rs2074735
rs61887421
rs7569755
rs2398113
rs13108245
rs964184
rs1384687
rs12462826
rs804281
rs11791258
rs6438900
rs1949633
rs3732220
rs142158911
rs8107974
rs10438978
rs2710651
rs7580771
rs3114045
rs12775091
rs635634
rs9409266
rs6672758
rs35270497
All − MR Egger
All − Inverse variance weighted
−10
0
10
20
MR effect size for
'Serum 25−Hydroxyvitamin D levels || id:ebi−a−GCST90000618' on 'Acne || id:finn−b−L12_ACNE'

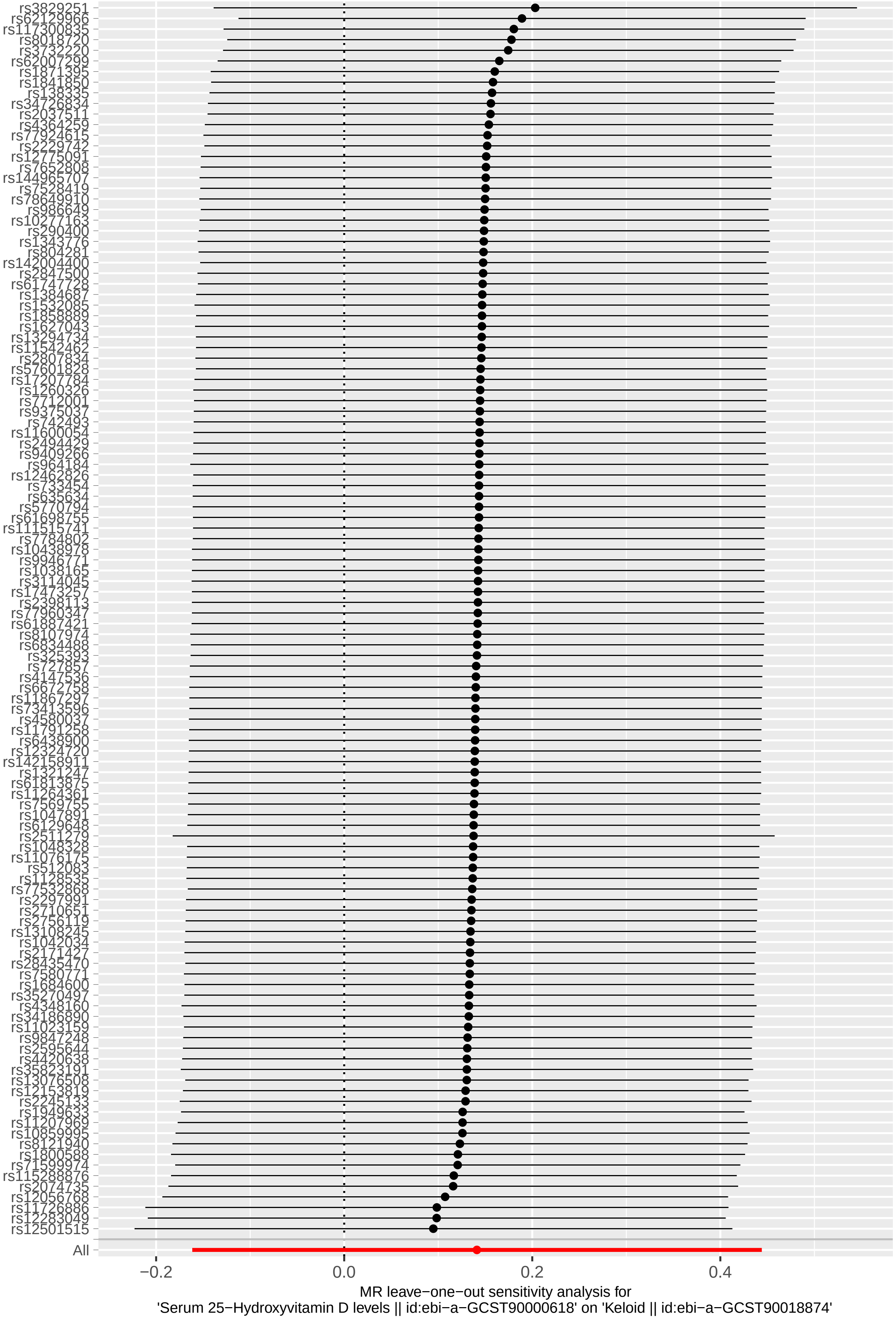
rs3829251
rs62129966
rs117300835
rs8018720
rs3732220
rs62007299
rs1871395
rs1841850
rs138335
rs34726834
rs2037511
rs4364259
rs77924615
rs2229742
rs12775091
rs7652808
rs144965707
rs7528419
rs78649910
rs986649
rs10277163
rs290400
rs1343776
rs804281
rs142004400
rs2847500
rs61747728
rs1384687
rs1532085
rs1858889
rs1627043
rs13294734
rs11542462
rs2807834
rs57601828
rs17207784
rs1260326
rs7712001
rs9375037
rs742493
rs11600054
rs2494429
rs9409266
rs964184
rs12462826
rs733454
rs635634
rs5770794
rs61698755
rs111515741
rs7784802
rs10438978
rs9946771
rs1038165
rs3114045
rs17473257
rs2398113
rs77960347
rs61887421
rs8107974
rs6834488
rs325393
rs727857
rs4147536
rs6672758
rs11867297
rs73413596
rs4580037
rs11791258
rs6438900
rs12324720
rs142158911
rs1321247
rs61813875
rs11264361
rs7569755
rs1047891
rs6129648
rs2511279
rs1048328
rs11076175
rs512083
rs1128535
rs77532868
rs2297991
rs2710651
rs2756119
rs13108245
rs1042034
rs2171427
rs28435470
rs7580771
rs1684600
rs35270497
rs4348160
rs34186890
rs11023159
rs9847248
rs2595644
rs4420638
rs35823191
rs13076508
rs12153819
rs2245133
rs1949633
rs11207969
rs10859995
rs8121940
rs1800588
rs71599974
rs115288876
rs2074735
rs12056768
rs11726886
rs12283049
rs12501515
All
−0.2
0.0
0.2
0.4
MR leave−one−out sensitivity analysis for
'Serum 25−Hydroxyvitamin D levels || id:ebi−a−GCST90000618' on 'Keloid || id:ebi−a−GCST90018874'

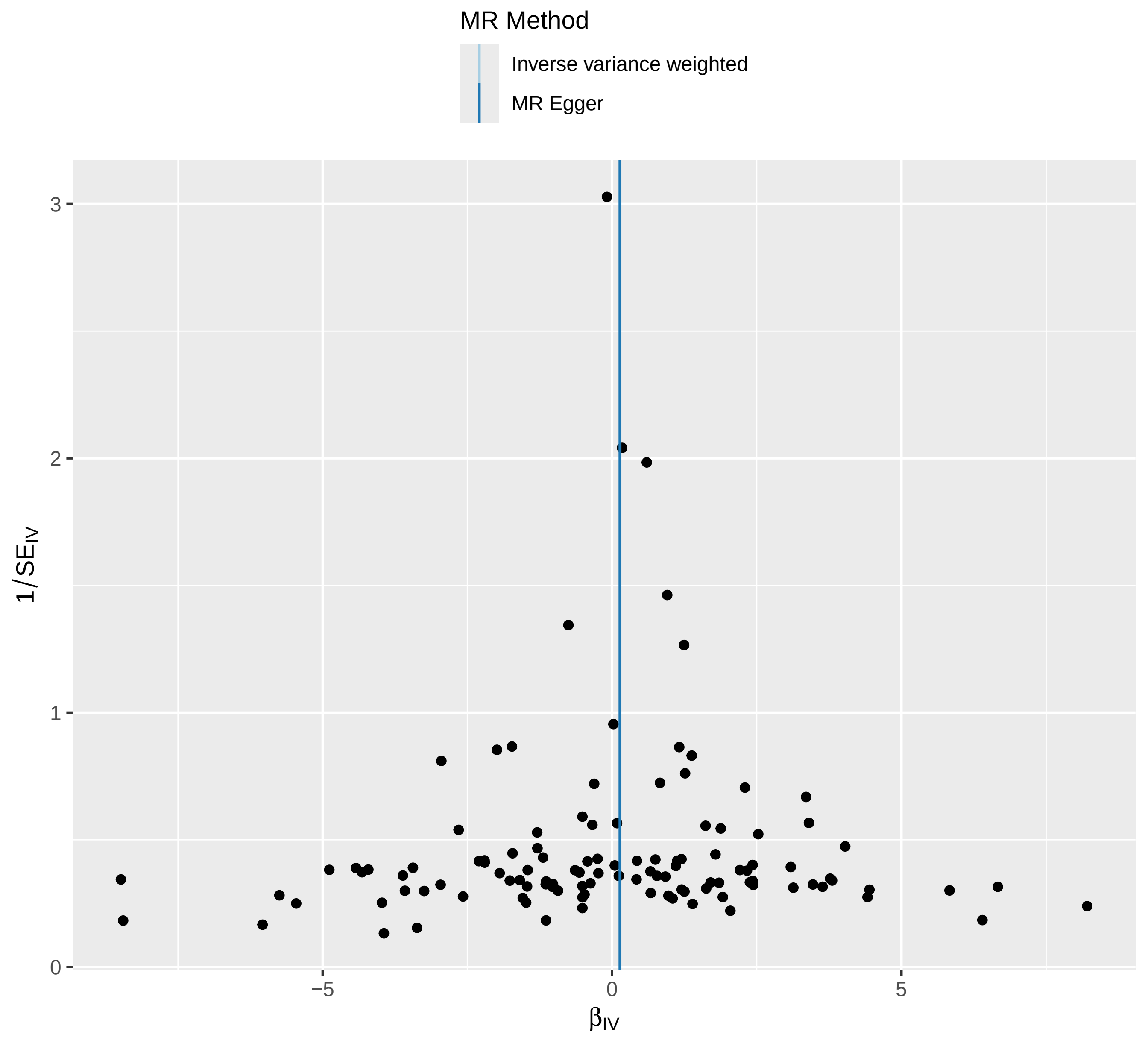

MR Method
Inverse variance weighted
MR Egger
3
2
1
0
−5
0
5
1/SE$_{IV}$
β$_{IV}$

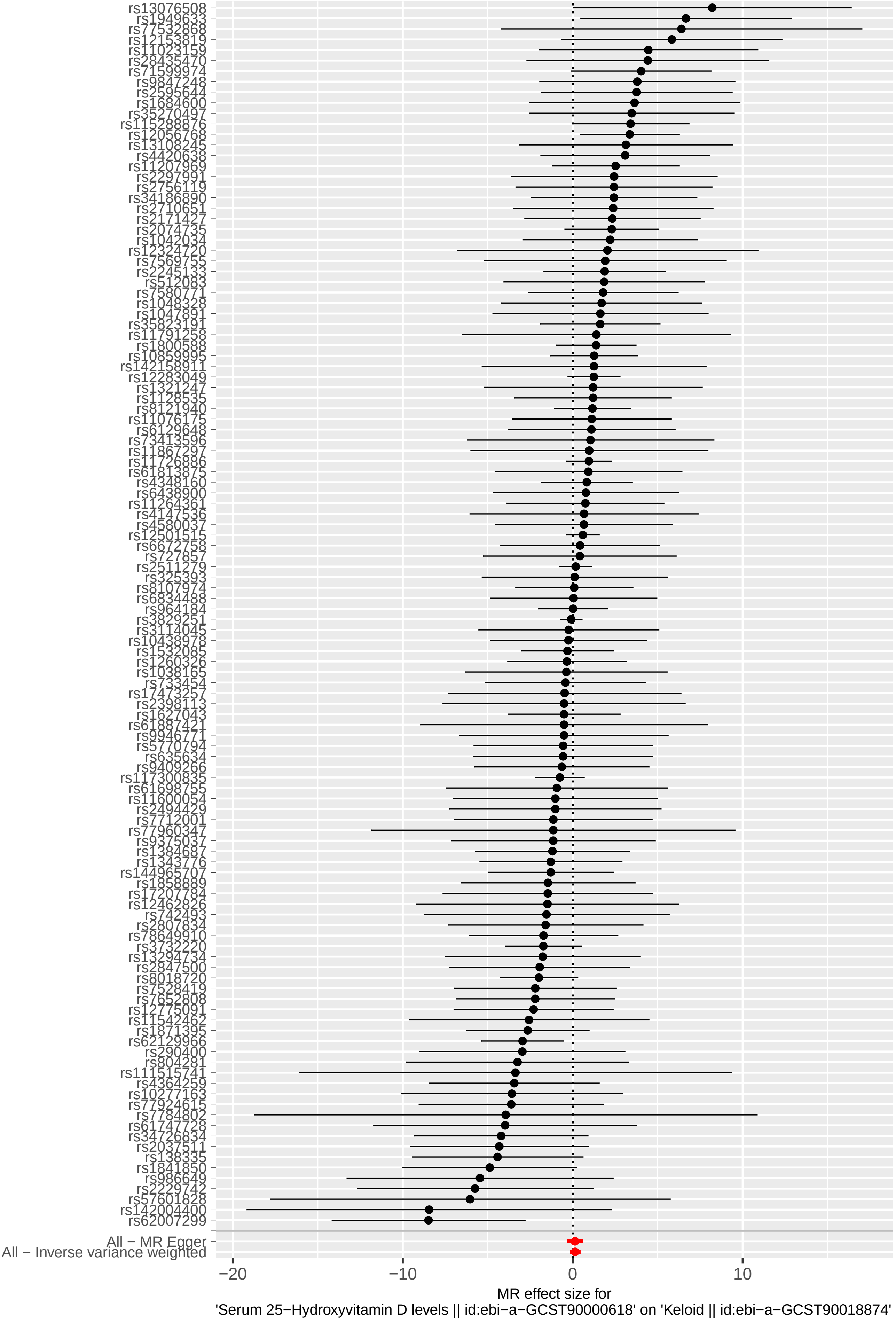

rs13076508
rs1949633
rs77532868
rs12153819
rs11023159
rs28435470
rs71599974
rs9847248
rs2595644
rs1684600
rs35270497
rs115288876
rs12056768
rs13108245
rs4420638
rs11207969
rs2297991
rs2756119
rs34186890
rs2710651
rs2171427
rs2074735
rs1042034
rs12324720
rs7569755
rs2245133
rs512083
rs7580771
rs1048328
rs1047891
rs35823191
rs11791258
rs1800588
rs10859995
rs142158911
rs12283049
rs1321247
rs1128535
rs8121940
rs11076175
rs6129648
rs73413596
rs11867297
rs11726886
rs61813875
rs4348160
rs6438900
rs11264361
rs4147536
rs4580037
rs12501515
rs6672758
rs727857
rs2511279
rs325393
rs8107974
rs6834488
rs964184
rs3829251
rs3114045
rs10438978
rs1532085
rs1260326
rs1038165
rs733454
rs17473257
rs2398113
rs1627043
rs61887421
rs9946771
rs5770794
rs635634
rs9409266
rs117300835
rs61698755
rs11600054
rs2494429
rs7712001
rs77960347
rs9375037
rs1384687
rs1343776
rs144965707
rs1858889
rs17207784
rs12462826
rs742493
rs2807834
rs78649910
rs3732220
rs13294734
rs2847500
rs8018720
rs7528419
rs7652808
rs12775091
rs11542462
rs1871395
rs62129966
rs290400
rs804281
rs111515741
rs4364259
rs10277163
rs77924615
rs7784802
rs61747728
rs34726834
rs2037511
rs138335
rs1841850
rs986649
rs2229742
rs57601828
rs142004400
rs62007299
All − MR Egger
All − Inverse variance weighted
−20
−10
0
10
MR effect size for
'Serum 25−Hydroxyvitamin D levels || id:ebi−a−GCST90000618' on 'Keloid || id:ebi−a−GCST90018874'
